# Functionality and Appearance of Sorghum‐Pearl Millet Composite Flour Fortified With Oyster Mushroom for Thin Porridges

**DOI:** 10.1002/fsn3.71630

**Published:** 2026-03-08

**Authors:** Alice Ndunge Charles, Monica Mburu, Daniel Njoroge, Mario Jekle, Viktoria Zettel

**Affiliations:** ^1^ Institute of Food Bioresources Technology Dedan Kimathi University of Technology Nyeri Kenya; ^2^ Department of Plant‐Based Foods, Institute of Food Science and Biotechnology University of Hohenheim Stuttgart Germany

**Keywords:** composite flours, *Pennisetum glaucum*, *Pleurotus ostreatus*, porridges, *Sorghum bicolor* (L) Moench

## Abstract

Porridges are the main staple food in sub‐Saharan Africa. They are generally starchy and are made from composite flours of various cereals. Evaluation of color, pasting, rheological, and functional properties of the composite flour samples was done, since these influence the cooking and product quality of the porridges. Sorghum‐pearl millet blends were substituted at levels from 10% to 50% using oyster mushrooms to form the composite flours. Rheometer and Rapid Visco Analyzer were used in the determination of rheological and pasting properties respectively, while Chroma Meter was used in color determination. Bulk density, water and oil absorption, and swelling qualities were also determined. The study findings indicated that fortified flours had a consistently higher water, oil, and swelling properties indicating that oyster mushrooms have a higher absorption capacity. The color parameters (L* (degree of lightness), b* (yellowness), chroma, hue angle) increased while the pasting parameters (peak, trough, breakdown, final, and setback viscosities) reduced with an increase in oyster mushroom levels; that is, final viscosity decreased with increased oyster mushroom levels in the pearl millet‐sorghum flours from 1072.7 ± 61.8 to 216.8 ± 21.2 mPas. For the dynamic rheological tests of the thin porridges prepared from the composite flours showed a distinctive gel‐like character under the measurement conditions, an indication that elastic behavior dominated over viscous behavior. The level of storage and loss moduli decreased with increasing substitution levels, which is coherent with the pasting properties. Research on physico‐functionality of sorghum‐pearl millet composite flours fortified with oyster mushrooms shows that the substitution of mushrooms has no significant negative effect on the composite flour functionality. Oyster mushrooms could therefore be used to fortify cereals without affecting their physical properties.

## Graphical abstract

This study investigated the functionality, color, pasting and rheological properties of sorghum–pearl millet composite flour fortified with oyster mushroom. Fortification increased water, oil and swelling capacities, while decreasing pasting viscosities. The color lightness and yellowness improved with increasing oyster mushroom levels. Rheological tests indicated gel‐like behavior (*G*′ > *G*″), showing enhanced elasticity of thin porridges. The findings demonstrate that oyster mushroom fortification can enhance cereal‐based porridge nutrition without compromising functional quality.

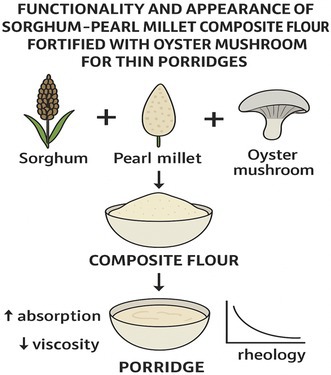

## 1 Introduction

In Sub‐Saharan Africa, the major staple foods are porridges type of products; thin gruels, stiff porridges, boiled doughs, steamed and boiled doughs which are generally made from cereals that is maize, millets and sorghum (Foster et al. 2020). For adults, thin porridge is a crucial breakfast and refreshing drink (Calvin and George 2018), while for children as complementary food (Kikafunda et al. 2006) and for the ill and the invalid as a source of nourishment (Wanjala et al., n.d.). Thin porridges locally known as *uji* in eastern Africa are made from flours of cereals and tubers; finger millet, maize, pearl millet, sorghum and cassava (Wanjala et al. 2016). They are made by addition of slurry in boiling water (10% to 20% w/v) with continuous stirring (Taylor and Emmambux 2008). Flour transformation into thin porridges is linked with irreversible physical starch alteration in excess of water. During pasting and retrogradation process complex fractal structures are formed after gelatinization, which results to the loss of the starch lamellar structure (Doutch et al. 2012). The thin porridges final color is influenced by the color of the flour used in its preparation (Calvin and George 2018). Sorghums and millets thin porridges or their composited flours with maize are usually light to dark brown in color with a hint of red (Ashworth et al. 1992; Wanjala et al. 2016). Quality factors such as desirability in flour are closely associated with its color and functionality. Therefore, when there in variations in color, the perception of consumers are likely to be impacted (Calvin and George 2018). The desirability of the thin porridges is not only affected by its appearance but also its viscosity. In many developing countries, infants are typically given cereal gruels with a high viscosity (and thus low energy density). This is because the gruels are diluted to reduce their viscosity (Araro et al. 2020; Makame et al. 2020). This has been viewed as a cause of low energy intake. It is suggested that a viscosity value of 1000–3000 cP is appropriate for the consistency of complementary foods, making them easy to swallow for infants and young children (Amagloh 2022; Araro et al. 2020; Mosha and Svanberg 1983).

The indigenous staple cereals used in making the porridges are generally starchy with limiting nutritional properties (Marcel et al. 2022). Several remedies through research efforts have suggested fortification of these starchy staple cereals with locally available and nutritious foods such as oyster mushroom due to its dense nutritional composition (Bamidele and Adebowale 2019; Bamidele and Fasogbon 2020; Ishara et al. 2018; Ng et al. 2017). To this end, there has been increased emphasis on the incorporation and utilization of oyster mushroom in formulation of these composite flours. Several studies have been devoted to how these foods can be prepared as composite to come up with safe and nutritious foods that are also acceptable (Bamidele and Fasogbon 2020; Siyame et al. 2021). Additionally, the findings from the first part of the research indicated that fortifying sorghum and pearl millet with oyster mushroom significantly enhanced the nutritional profile of the composite flours, highlighting their potential contribution toward alleviating protein energy malnutrition and micronutrient deficiencies (Ndunge Charles et al. 2024). The choice of sorghum and pearl millet in the research was due to the fact that they are staple cereals and, therefore, commonly used as main vehicles in the fortification programs. Additionally, they are considered smart‐climate crops (Pixley et al. 2023). Although as a fortificant, oyster mushroom improved the nutritional composition of these cereals, there was a great decrease in the consumer acceptability of the fortified flours, which might have been due to the effect of oyster mushroom on the techno‐functionality of the flours.

Therefore, when it comes to food processing, research on pasting and rheological properties of food systems has a crucial contribution enabling the production process as well as the stability and flavor of the final products. In addition, rheological characteristics are also crucial in designing flow processes and optimizing recipes, measurement of processing and storage stability, texture prediction, conformational changes, and molecular understanding in food ingredients (Dasa and Binh 2019). Rheology explains how materials react to applied stress and deformation (Chen et al. 2010). Previous studies have shown that thin porridges from sorghum and pearl millet have high viscosity, although the addition of oyster mushroom might have a negative effect on viscosity, which might be attributed to its dilution effect due to a decrease in carbohydrate content and an increase in fiber and protein content. As the previous research results indicate a decrease in consumer acceptability, these greatly explain the effect of oyster mushrooms on these properties. Therefore, analyzing pasting, functional, and rheological properties is crucial in product formulation and its acceptability. In addition, evaluating the color of the composite flour is of great importance, as it has a direct relation to the consumer's perception of the thin porridges.

However, there are limited studies/information on the pasting behavior and functionality of oyster mushroom cereal‐based composite flours, as well as the rheological properties of thin porridges. Therefore, this research intended to investigate the color, pasting, functional properties of composite flours and rheological properties of the composite flour thin porridges from sorghum‐pearl millet flour fortified with oyster mushroom, in order to understand the physico‐functionality of these flours, which affects both the eating and cooking quality.

## 2 Materials and Methods

### 2.1 Materials

Raw materials that is red sorghum (5 kg) and pearl millet (5 kg) were purchased from Nyeri local market, Kenya while oyster mushrooms were obtained from a farmer in Kiambu county, Kenya. Sunflower oil was obtained in a local supermarket (REWE Markt GmbH, Cologne, Germany) Paraffin oil was obtained via a laboratory supplier (Carl Roth GmbH+Co. KG, Karlsruhe, Germany). Distilled water was obtained from the lab.

### 2.2 Materials Preparation

Pearl millet and sorghum were first sorted separately, washing of the cereals was then done followed by soaking in water for anti‐nutrient reduction for about 19 ± 1 h at room temperature. After soaking, pearl millet and sorghum were thoroughly rinsed with tap water then dried with a solar dryer with temperatures ranging between 46.5°C ± 3.5°C (Afify et al. 2012). Oyster mushrooms were sliced, then solar dried separately in the solar drier together with the grains.

### 2.3 Flour Formulation

An ultra‐centrifugal mill ZM 200 (Retsch GMbH, Haan, Germany) was used in the milling of the dried raw materials (oyster mushrooms, pearl millet and sorghum) separately, using a 0.250 mm sieve at 2504.32g. The composite flour blends (CFB) formulation was done by first making a mixture of sorghum and pearl millet in the ratio 50:50 to make the control (the choice was based on what is in existence traditionally), then different percentages composite flour formulation of oyster mushroom with the control (pearl millet‐sorghum) were made as presented in Table 1. The CFB were produced individually for each measurement, that is each component was first weighed and then blended. This was necessary in order to ensure that the desired proportions of the individual components were always present in the respective sample and to address the biological variability that is also visible due to the standard deviations for the individual measurements. Each analytical procedure was then performed three times, so that in total 9 measurement values were obtained. Formulation's ratios selection was based on finding the optimal nutrient rich blend and that will not have adverse effect on the sensory evaluation parameters of thin porridges prepared from composite flours as well as their functionality. Similar research on flours formulations of oyster mushrooms with other cereal‐based foods were used as reference (Bamidele and Fasogbon 2020; Ekunseitan et al. 2017; Ishara et al. 2018; Siyame et al. 2021).

**TABLE 1:** Blending percentage ratios of oyster mushroom, red sorghum and pearl millet composite flour blends (CFB) with the numbers indicating the oyster mushroom percentages used.

Treatment codes	Blending proportions		
	Red sorghum (%)	Pearl millet (%)	Oyster mushroom (%)
Control (CFB0)	50	50	0
CFB10	45	45	10
CFB20	40	40	20
CFB30	35	35	30
CFB40	30	30	40
CFB50	25	25	50

**TABLE 2:** Pasting properties of sorghum‐pearl millet and oyster mushrooms composite flours.

Sample	PV (cP)	TV (cP)	BV (cP)	FV (cP)	SV (cP)	Stability ratio	Setback ratio	PT (min)	PT (°C)
Control (CFB0)	655.3 ± 18.0^a^	633.0 ± 21.2^a^	22.33 ± 3.51^a^	1072.7 ± 61.8^a^	439.7 ± 41.0^a^	0.97 ± 0.10^a^	1.70 ± 0.06^a^	6.85 ± 0.12^a^	89.83 ± 0.4^a^
CFB10	281.67 ± 13.05^b^	265.67 ± 13.32^b^	16.0 ± 1.73^ab^	472.3 ± 19.4^b^	210.67 ± 8.62^b^	0.94 ± 0.22^b^	1.79 ± 0.10^a^	6.76 ± 0.17^a^	92.59 ± 2.51^a^
CFB20	207.5 ± 30.9^c^	193.3 ± 28.8^c^	14.25 ± 2.06^a^	368.8 ± 52.6^c^	175.5 ± 23.8^bc^	0.93 ± 0.04^b^	1.91 ± 0.13^a^	6.87 ± 0.10^a^	92.81 ± 1.02^a^
CFB30	181.0 ± 20.1^c^	165.0 ± 14.53^cd^	16.0 ± 6.08^b^	326.3 ± 37.4^cd^	161.3 ± 23.0^c^	0.92 ± 0.13^bc^	1.97 ± 0.62^ab^	6.85 ± 0.12^a^	92.43 ± 1.07^a^
CFB40	145.33 ± 17.21^d^	135.33 ± 14.01^de^	10.0 ± 5.0^b^	272.0 ± 41.4^de^	136.7 ± 28.0^c^	0.91 ± 0.12^c^	2.0 ± 0.43^b^	6.81 ± 0.20^a^	92.58 ± 2.04^a^
CFB50	133.50 ± 6.61^d^	122.50 ± 4.12^e^	11.0 ± 4.6^b^	216.8 ± 21.2^e^	94.25 ± 19.77^d^	0.91 ± 0.08^c^	2.01 ± 0.30^b^	6.606 ± 0.32^a^	92.38 ± 2.17^a^

*Note:* Values are means ± SD and values in the same column with different superscript letters differ significantly from each other (*p* < 0.05).

Abbreviations: BV, breakdown viscosity; cP, centipoise; FV, final viscosity; PT (°C), peak temperature; PT (min), peak time; PV, peak viscosity; setback ratio, ratio of final viscosity to hold viscosity; stability ratio, ratio of hold viscosity to peak viscosity; SV, setback viscosity; TV, trough viscosity.

### 2.4 Methods

#### 2.4.1 Pasting Properties

Rapid Visco Analyzer (RVA; RVA‐4 Newport scientific PVT Ltd., Australia) was used in pasting characteristics determination of the formulated blends, using AACC method 76‐21.02 as stated by Alemneh et al. (2022). 3 g of the sample (corrected to 14% moisture content) and 25 mL of distilled water were used for the measurement. After inserting the sample into the measuring canister, it was roughly pre‐mixed with the plastic paddle by hand before it was inserted in the RVA. The slurry heating was then done from 50°C to 95°C and cooling was done back to 50°C in a span of 13 min, rotating of canister was done at 160 rpm and continuously stirred using a plastic paddle (compare STD2 profile of the RVA). Pasting characteristics measured included trough, peak, setback, break down, final viscosity, pasting temperatures and time from pasting profile which were read using computer's thermocline for windows (TCW3) software.

#### 2.4.2 Bulk Density

Each sample's bulk density (BD) was evaluated using the method described by Adegbanke and Ilesanmi (2018). 50 g of flour by weight was filled into a glass measuring cylinder (100 mL). After that, repeated tapping of the cylinder on the lab bench was done until volume remained constant and then the change in volume was recorded. Afterwards, BD (g/mL) was calculated using Equation (1):

(1) $$\text{Bulk density}\;\left(g/\mathrm{mL}\right)=\text{sample weight}\;\left(g\right)/\text{sample volume}\;\left(\mathrm{mL}\right)$$

#### 2.4.3 Water and Oil Absorption Capacity

As described by Adegbanke and Ilesanmi (2018), water absorption capacity (WAC) and oil absorption capacity (OAC) were analyzed. Exactly 1 g of flour sample was combined with 10 mL of distilled water (for WAC) or sunflower oil (for OAC) and mixed thoroughly for 2 min using a glass rod. Centrifugation of the obtained suspension was done for 30 min at 479.45g, and measuring of the obtained supernatant was then done by the use of a 10‐mL graded cylinder. Densities of the water and sunflower oil considered were 1.0 g/cm^3^ and 0.96 g/mL respectively. Then, WAC and OAC were calculated using Formula (2) and (3) respectively:

(2) $$\text{Water absorption capacity}\;\left(\mathrm{mL}/g\right)=\frac{\text{volume of water used}-\text{volume of water}\;\text{after centrifugation}\;\left(\mathrm{mL}\right)}{\text{weight of sample}\;\left(g\right)}$$

(3) $$\text{Oil absorption capacity}\;\left(\mathrm{mL}/g\right)=\frac{\text{volume of oil used}-\text{volume of oil after centrifugation}\;\left(\mathrm{mL}\right)}{\text{weight of sample}\;\left(g\right)}$$

#### 2.4.4 Swelling Index

The amount of water‐soluble solids per unit weight of the sample is swelling index (SI). Weighing approximately 3 g of each flour sample and transferring it into a 50‐mL graded cylinder was done. The initial volume was read after adding the water to the CFB sample before mixing. Then the CFB and the water were mixed with 30 mL distilled water. After swirling, the measuring cylinder was left to stand for 1 h at room temperature (Awolu 2017). The volume change was recorded and later the SI calculation was done using Formula (4):

(4) $$\mathrm{SI}\;\left(\mathrm{mL}/g\right)=\frac{\text{volume after soaking}-\text{volume before soaking}\;\left(\mathrm{mL}\right)}{\text{original weight of sample}\;\left(g\right)}$$

#### 2.4.5 Swelling Capacity

Swelling capacity (SC); the obtained gel from analysis of swelling index (substance left over after discarding the supernatant) was used in flour sample's swelling capacity calculations using Formula (5):

(5) $$\text{Swelling capacity}\;\left(g/g\right)=\frac{\text{weight of}\;\mathrm{wet}\;\mathrm{gel}\;\left(g\right)}{\text{weight of sample}\;\left(g\right)}$$

#### 2.4.6 Thin Porridges Preparation

Thin porridge preparation was made from each of the composite flour blends as presented in Table 1 using the method described by Onabanjo et al. (2008) with slight modification. Approximately 50 g of the composite flour sample was combined with 100 mL of cold water in a small bowl to form a slurry and then set aside. Afterwards, 450 mL of water was brought to a boil in an aluminum pan and then the previously prepared slurry was added to the boiling water to achieve a ratio (1:11 w/v). The thin porridges were then cooked at 90°C for 5 min with constant stirring to prevent them from sticking to the pan and coagulating; thereafter, the porridges were taken out of the heating medium. Dynamic rheological tests were done thereafter.

#### 2.4.7 Color Analysis

Determination of the formulated composite flours color characteristics (L*a*b*) was done by the use of a Chroma Meter (Konica Minolta CR‐300, Osaka, Japan) according to Dhillon et al. (2023). The chroma meter was first calibrated using a white standard and then the composite flours color measurements were taken based on the following parameters: L* lightness (100 white, 0 black), −b* (blueness) and + b* (yellowness), −a* (greenness), +a* (redness). Subsequently, the values of hue and chroma were calculated using the Formula (6) and (7).

(6) $$\text{Chroma}=a^{*2\;}+b^{*2}$$

(7)

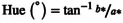




#### 2.4.8 Dynamic Rheological Tests

A rheometer (Physica MCR 302, Anton Paar GmbH, Graz Austria Europe) was used in rheological properties measurement of the composite flours. Thin porridge was first prepared according to Onabanjo et al. (2008). A thin porridge sample was scooped using a spatula and transferred onto the rheometer lower plate and pressed down using the upper plate (diameter of 25 mm), until a 2 mm gap was obtained. A razor was used to trim off the excess of the sample that protruded beyond the edge of the upper plate, and drops of paraffin fluid were applied around the uncovered surface to avoid drying or moisture loss during the test. The sample was then covered around with a lid. Controlled by an external thermostatic bath, at a constant temperature of 30°C on the lower plate, all the dynamic rheological tests were done.

The samples were initially subjected to amplitude sweeps. The purpose of amplitude sweeps primarily is determination of LVE range (linear viscoelastic region) limit. The energy of deformation stored by the samples during the shear process is the storage modulus (*G*′); it indicates samples' elastic behavior. Loss modulus (*G*″) measures energy deformation utilized by the sample during shear process and represents the viscous behavior of a sample (Ketut Oscar Edy 2010). The thin porridges were subjected to amplitudes from 0.01% to 101%, maintaining an angular frequency (*ω*) of 10 rad/s (Mezger 2011).

The amplitude of the LVE was determined for all thin porridge samples. With a constant amplitude value and changing frequencies the oscillatory test that investigates time dependent shear behavior is the frequency sweep (Ketut Oscar Edy 2010). Gels at low frequency behave like a fluid and at high frequency as solid. Fluids to solid transition‐like behavior happens at a critical frequency that equals the inverse time of relaxation (Ketut Oscar Edy 2010). In this study oscillation frequency application ranging from within 0.1 to 10 Hz at constant strain *γ* = 1 each logarithmic frequency decade equated to 30 measurement points.

The dynamic rheological tests were made in 3 replicates and each time a newly prepared sample was made making a total of 9 tests per sample. The rheological viscoelastic parameters determined were the storage modulus *G*′ and the loss modulus *G*″.

### 2.5 Statistical Analysis

All the analyses were conducted in triplicate. The findings were expressed as mean ± standard deviation. The data were statistically analyzed using the one‐way analysis of variance (ANOVA). Fishers LSD test was used in means separation of the results obtained with 5% level of significance, using Minitab 18.1 statistical software.

## 3 Results and Discussion

### 3.1 Pasting Properties of Sorghum‐Pearl Millet and Oyster Mushroom Composite Flours

The fortified flours' pasting parameters are presented in Table 2. Wang et al. (2018), reported that when heated, starch granules hydrate, expand, and form a paste. The granular starch structure collapses when crystallites melt, double helices unravel, and hydrogen bonds dissolve. This modification, known collectively as gelatinization, is accompanied by the distinctive birefringence loss of organized granules. During cooling, the disaggregated starch chains retrograde into a more organized structure that is different from original granules.

Peak viscosity (PV) is known as starch's potential to freely expand before breaking down physically (Eke et al. 2010). The PV of the composited samples decreased with increase in oyster mushroom percentages, in which control had the highest peak viscosity (655.3 cP), while composited flour with 50% oyster mushroom had the lowest (133.50 cP). According to Ishara et al. (2018) upon addition of oyster mushroom, the carbohydrate content decreases while the protein content increases; therefore, this phenomenon might have also been the reason for PV reduction. In addition, the previous results of the research indicated that, upon addition of oyster mushroom to the cereals, there was a dilution effect, which might have been attributed to the high protein content in the mushrooms and low carbohydrate content (Ndunge Charles et al. 2024). Therefore, the protein present competed with starch granules for water, thus leading to a decreased viscosity. This phenomenon explains why the composite flour with the highest amount of protein had the lowest viscosity. However, not only the carbohydrate content, but also the composition of the carbohydrates, for example, the mushroom sugar trehalose, affects the pasting of starch (Su et al. 2024).

Trough viscosity (TV) is known as hot paste viscosity, shear thinning, or holding strength because of viscosity breakdown that occurs. It assesses the paste's ability to survive disintegration while cooling. The viscosity explains the lowest point at which a sample cools down following the initial peak. Upon an increase in oyster mushroom level, the flour samples' TV decreased (control 633.0 cP to 122.5 cP for CFB50). Composite flours holding strength is the lowest viscosity after peak that allows flours' starch granules to remain undisturbed throughout a holding period of constant temperature, time and shear stress (Ekunseitan et al. 2017; Otegbayo et al. 2006).

At a holding temperature of 95°C, the stirring caused the granules to rupture and fragment, resulting in a reduction in viscosity after it had reached its maximum. This is known as breakdown viscosity (BV), which is a tendency of the enlarged starch granules to rupture/disintegrate continuously stirred and when held at high temperatures (Gayin et al. 2009). BV indicates paste stability (Ohizua et al. 2017). Control sample BV was (22.33 cP) while composite flour with 50% oyster mushroom had 11.0 cP, an indication of a decrease in the viscosity with increment in the amount of oyster mushroom. A lower breakdown viscosity indicates a lesser ability to preserve viscosity and structure because it indicates that the flours starch is breaking down more quickly when heated.

The starches' ability to solidify into a viscous paste after being cooled is known as final viscosity (FV) (Eke et al. 2010; Ohizua et al. 2017; Otegbayo et al. 2006). Arinola et al. (2016) reported that FV is used to predict and define starchy foods' final textural quality. It also gives the measurement of paste resistance to shear force when stirred (Eke et al. 2010). This indicates that composite flour with 50% oyster mushroom (CFB50) becomes unstable after cooling since it has the lowest final viscosity (216.8 cP).

Setback viscosity (SV) occurs in the pasting curve after starch has cooled and it involves reassociation, retrogradation or reordering of starch molecules. Effah‐Manu et al. (2022) suggested that low setbacks in flours might be linked to reduced content of amylose which has a high molecular weight. According to Ikegwu et al. (2010), products made from such flour exhibit higher setback values when cooled, possibly due to the abundance of amylose which is more susceptible to retrogradation compared to amylopectin. In addition, CFB50 had the lowest setback viscosity, an indication of low amount of starch and therefore the starch present is less able to regain thickness and its original structure after cooling, which agrees with the previous proximate results which showed that the flour had the lowest carbohydrate content (Ndunge Charles et al. 2024). Setback viscosity decrease was also reported as levels of oyster mushroom increased in the fortification of wheat and pearl millet flour (Mahajan et al. 2015).

According to Adebowale et al. (2011), pasting time measures the time taken to cook starch. The cooking time of all the composite ratios did not significantly (*p* < 0.05) differ; therefore, it indicates that cooking time for all the flour samples was within a range of approximately 6 min. Pasting temperature is the temperature at which the first discernible rise in viscosity is recorded; it is an index that represents the initial change caused by starch granules swelling, and it is used in measuring the minimal temperature required in cooking a certain food (Arukwe 2021). An increase of pasting temperature was noted on increasing oyster mushroom flour, although no significant (*p* < 0.05) change was noted. High pasting temperatures indicate higher gelatinization tendency, which means the flour requires higher temperatures for the initiation of gelatinization, water binding capacity, and also lowers starch's flour swelling qualities because of higher degree of association within the granules of starch (Adebowale et al. 2011; Arinola et al. 2016; Ikegwu et al. 2010). The pasting temperature increment may be attributed to the increased fiber and protein in the fortified flour that competed with starch for water molecules (Ndunge Charles et al. 2024). This caused a delay in starch gelatinization that led to increased pasting temperature in the fortified flours. This phenomena was also reported by (Mahajan et al. 2015) in which an increase in pasting temperature was recorded upon fortification of wheat and pearl millet with oyster mushroom.

Addition of oyster mushrooms to sorghum‐pearl millet flour (control) influenced the pasting profile of the fortified flours, where its inclusion led to a significantly (*p* < 0.05) decrease in all pasting characteristics (PV, TV, BV, FV, SV) except temperature which increased while no significant time change was observed. The increased mushroom flour fortification led to increased fiber and protein which led to an increased competition with starch molecules for water thus causing decreased capacity of hydration of starch as observed by (Mahajan et al. 2015). This phenomenon might have had a contribution to a decrease in all the viscosities. The results agree with those of Bamidele and Adebowale (2019) and Ng et al. (2017), who also noted reduction in pasting properties of sorghum‐mushrooms composite flour and wheat oyster mushroom blends. This might be due to the high amount of small sugars in mushrooms, like trehalose, that affect pasting of starch (Su et al. 2024).

### 3.2 Functional Properties of Sorghum‐Pearl Millet and Oyster Mushroom Composite Flours

#### 3.2.1 Bulk Density

The composited samples' functional properties are presented in Table 3. The mass of particles which occupies a certain space or volume is what is referred to as bulk density (Djamila et al. 2020). It is an important element that contributes to the determination of how raw materials are handled and packaging requirements (Ajanaku et al. 2012). The BD findings of the flour blends differed significantly (*p* < 0.05) from control except for the flour sample CFB10. CFB10 did not differ significantly from flour CFB20 while CFB20, CFB30, and CFB40 were not significantly different (*p* < 0.05); however, CFB50 was significantly different from all the formulated flours. BD of the samples ranged from (control) 0.54–0.47 g/mL (CFB50), showing a reduction in bulk density upon increment of oyster mushrooms quantity. The findings corroborate with those of Ishara et al. (2018), which showed that BD of oyster and button mushroom substitution in maize decreased upon addition of mushroom flour up to 50%, where the values for oyster mushroom substituted in maize flours being within the same range (0.53–0.35 g/mL) as this study. The decrease in the bulk density may be attributed to the high porous, lightweight, and less compact structure of oyster mushrooms as compared to the grains (Thirumuruga Ponbhagavathi et al. 2022). Additionally, increasing the oyster mushroom level led to the loosening of the starch polymers due to the dilution effect created by increasing protein content and decreasing carbohydrate content as reported in the previous results by Ndunge Charles et al. (2024), significantly influencing the low BD.

**TABLE 3:** Functional properties of sorghum‐pearl millet and oyster mushroom composite flours.

Functional properties	BD (g/mL)	WAC (mL/g)	OAC (mL/g)	SI (mL/g)	SC (g/g)
Control (CFB0)	0.54 ± 0.01^a^	0.95 ± 0.05^d^	0.43 ± 0.06^e^	0.95 ± 0.04^e^	2.83 ± 0.08^d^
CFB10	0.53 ± 0.02^a,b^	1.03 ± 0.05^d^	0.62 ± 0.06^d^	1.04 ± 0.09^d,e^	3.09 ± 0.2^c^
CFB10	0.52 ± 0.00^b,c^	1.27 ± 0.01^c^	0.72 ± 0.05^c^	1.15 ± 0.01^d^	3.25 ± 0.07^b^
CFB30	0.51 ± 0.00^c^	1.39 ± 0.05^b^	0.78 ± 0.01^b,c^	1.41 ± 0.1^c^	3.35 ± 0.01^b^
CFB40	0.50 ± 0.00^c^	1.53 ± 0.06^a^	0.86 ± 0.06^a,b^	1.72 ± 0.09^b^	3.65 ± 0.00^a^
CFB50	0.47 ± 0.02^d^	1.59 ± 0.06^a^	0.93 ± 0.05^a^	2.24 ± 0.2^a^	3.78 ± 0.05^a^

*Note:* Mean values (*n* = 3) ± SD, values within the same column with different letters significantly (*p* < 0.05) differ.

Abbreviations: BD, bulk density; OAC, oil absorption capacity; SC, swelling capacity; SI, swelling index; WAC, water absorption capacity.

#### 3.2.2 Water Absorption Capacity

Water absorption capacity (WAC) is the ability of a product to associate with water under limiting conditions. The WAC of the formulated blends ranged between 0.95 and 1.59 mL/g. Water absorption capacity increased with increment in oyster mushroom substitution. The control (CFB0) had the lowest (0.95 mL/g) and CFB50 had the highest WAC with 1.59 mL/g. CFB0 and CFB10 composite flour were not significantly at *p* < 0.05 different, CFB20 and CFB30 were significantly different (*p* < 0.05) and from all the other flours. However, the CFB40 and CFB50 flours did not differ significantly. The increment in WAC may be due to the increment in the amount of protein upon increment in the oyster mushroom percentages as reported earlier (Ndunge Charles et al. 2024). The protein content led to an increase in the polar amino acids (i.e., lysine) which have high hydrophilic sites thus increasing water binding through hydrogen bonding. Additionally, oyster mushroom are rich in dietary fiber as reported in the previous results Ndunge Charles et al. (2024), which have high water affinity as it acts as hydrophilic material thus increasing the flours capacity to absorb water molecules. The present findings are in agreement with others in literature that have shown that WAC increases with increased oyster mushroom amounts in composite flours with maize flour (Ishara et al. 2018; Tersoo‐Abiem et al. 2019). Similar effects of increment in the WAC was reported by Abou‐Zaid et al. (2012) when mushroom micelles were substituted with wheat flour. The increased WAC in this research could also be due to the drying methods of the raw materials especially oyster mushroom as solar dying could cause increase in the cell elasticity and thus increased absorption capacity of the flours (Djamila et al. 2020).

#### 3.2.3 Oil Absorption Capacity

Flour's ability to maintain flavor retention, which is vital in the formulation of food, is the oil absorption capacity (OAC). Oil absorption of food gives it flavor and soft texture; therefore, contributing to improvement of mouthfeel and flavor retention. In this study, the oil absorption of the composite flours increased with the increment in the percentage of mushrooms. Control had the lowest OAC value (0.43 mL/g), which was significantly different from all others shown in Table 3. A similar effect upon substitution of millet with mushroom flour had been previously reported, although the values of the OAC were slightly higher, ranging from 1.33 to 1.95 mL/g (Tersoo‐Abiem et al. 2019). The increment of OAC may be due to the presence of more hydrophobic amino acids (leucine), which shows superior lipid binding sites, and this may be the reason why flours with a high amount of oyster mushroom, which is abundant in protein, have a high value of OAC (Ishara et al. 2018). However, flour blends with lower OAC have a higher ability to retain flavor (Oladele and Aina 2007), this also is in line with the research sensory evaluation results obtained by the previously published study of Ndunge Charles et al. (2024).

#### 3.2.4 Swelling Index and Swelling Capacity

Swelling capacity (SC) and Swelling index (SI) of the composited blends increased upon increased mushroom percentages. The values of SI ranged from 0.95 to 2.24 mL/g. Nonetheless, SI of CFB10 did not differ significantly from control and CFB20, while CFB20, CFB30, CFB40 and CFB50 were significantly different (*p* < 0.05). The values of SC ranged between 2.83 and 3.78 mL/g. The control had the lowest SC with 2.83 mL/g while CFB50 had the highest (3.78 mL/g). However, SC of the control and CFB10 differed significantly from each other and from the other composite flours, while CFB20, CFB30 were not significantly different. CFB40 and CFB50 were not significantly different. The SI and SC increase in the flours may be due to the hydrophilic constituents of the raw materials for example the protein and polysaccharides which relates to the water affinity and diffusion phenomena (Ishara et al. 2018). Similar effects were observed in substitution of maize flour with oyster mushroom in which both SC and SI of the fortified flours increased, probably due to fibers present and the porous shape that enhanced water retention, absorption and swelling of the flours particles in water (Ishara et al. 2018). Similarly, substitution of millet with oyster mushroom had a similar trend of increase in the SC although the reported values were slightly lower (2.17–3.78 g/g) (Tersoo‐Abiem et al. 2019). A higher product's SC has shown to have good textural and flow properties.

**FIGURE 1:**
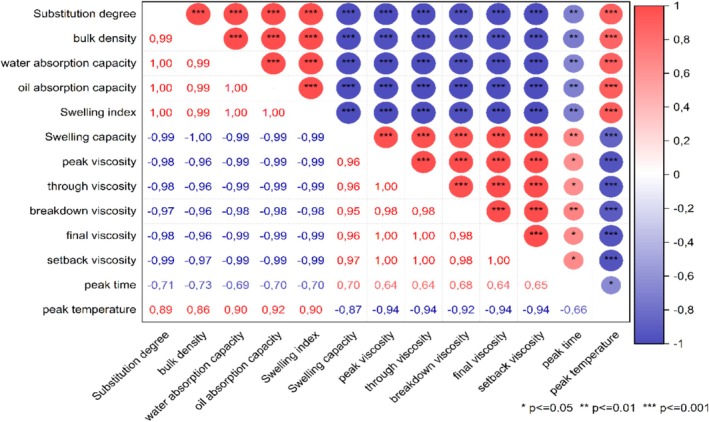
Correlation analysis of composite flour blends substitution levels and pasting properties as well as techno‐functional properties.

To evaluate all composite flour blends samples together a correlation analysis was performed. The results are presented in Figure 1. Bulk density, water absorption capacity, oil absorption capacity and swelling index are highly significant positively correlated with the substitution levels within the composite flour blends. For the pasting properties the correlation is highly negatively correlated. This might be due to the mushroom originated trehalose, which results in a liquification of the porridge perception as also stated in Ndunge Charles et al. (2024). Only the pasting temperature is positively correlated with increasing oyster mushroom. It is, however, also positively correlated with the techno‐functional parameters of the composite flour blends.

### 3.3 Color

Color is a crucial attribute of quality that determines consumer preferences of any product (Dhillon et al. 2023). From the results in Table 4, it was noted that L* as well as b* values of all formulated composite flour blends increased with increase in percentages of the mushroom flour, while the a*values reduced with increment in mushroom. To be specific, L* values ranged from (control) 73.47–80.00 (CFB50), a* values ranged from (control) 3.00–0.75 (CFB50) while the b* values ranged from (control) 9.65–15.85 (CFB50). It was noted that the a*, b* values, chroma and hue angle values of all the formulated ratios were significantly different (*p* < 0.05). All a* values of the flour blends differed significantly (*p* < 0.05), except CFB40 and CFB50 which did not show any significant difference from each other.

**TABLE 4:** Color characteristic of sorghum‐pearl millet and oyster mushroom composite flours.

Sample	L* (0 black, 100 white)	a* (+a* redness, −a* greenness)	b* (+b* yellowness, −b* blueness)	Chroma	Hue (°)
Control (CFB0)	73.47 ± 0.41^e^	3.00 ± 0.22^a^	9.65 ± 0.28^f^	10.11 ± 0.28^d^	72.73 ± 1.23^f^
CFB10	75.03 ± 0.67^d^	2.47 ± 0.17^b^	11.20 ± 0.14^e^	11.47 ± 0.11^e^	77.53 ± 0.97^e^
CFB20	76.30 ± 0.76^c^	1.95 ± 0.22^c^	13.00 ± 0.41^d^	13.15 ± 0.40^d^	81.45 ± 1.09^d^
CFB30	78.26 ± 0.58^b^	1.68 ± 0.16^d^	13.72 ± 0.42^c^	13.82 ± 0.41^c^	83.02 ± 0.70^c^
CFB40	79.41 ± 0.45^a^	1.26 ± 0.14^e^	14.41 ± 0.42^b^	14.47 ± 0.41^b^	84.99 ± 0.59^b^
CFB50	80.00 ± 0.42^a^	0.75 ± 0.31^f^	15.85 ± 0.39^a^	15.87 ± 0.40^a^	87.29 ± 1.12^a^

*Note:* Mean values (*n* = 5) ± SD, values within the same column with different letters significantly (*p* < 0.05 differ). L* value indicates degree of lightness.

All the flour samples had positive a* values indicating that all samples were more reddish, but the red color as seen in Table 4 decreased upon increment of oyster mushroom flour, but the level of reddish color differed significantly for all the samples (*p* < 0.05). Red color decrease could be due to the decrease in the levels of sorghum flour upon substitution with the oyster mushroom, which tends to be more greenish than reddish. Sorghum contains a flavonoid pigment called apigeninidin, which is reddish‐orange in color and therefore might have contributed to the reddish color (Zannou et al. 2025).

The L* values of the flour blends upon addition of oyster mushroom were moving toward 100 indicating that the flours were becoming brighter. The b* values of all the formulated flours were positive, meaning that they were yellowish in color; the intensity of the yellow color increased upon addition of a higher percentage of oyster mushroom and the level of yellowish differed significantly (*p* < 0.05). The increase in the yellow color upon increase in oyster mushroom might have been attributed to the yellowish color pigment known as pheomelanin found in the mushrooms caps (Zhang et al. 2022).

Color that is perceived by the naked eye and measured in degrees is the hue angle (Desalegn and Hailu 2020). Hue angles, just like chroma, increased with increment in mushroom levels; the hue angle values ranged from CFB0 (control) 72.73° to 87.29° (CFB50). The hue angles that are greater than 90° denote yellow color, whereas those lower than 90° suggest a slightly yellowish color, as noted in all the flour samples. Therefore, the measured values placed all flour samples in the yellow region of the CIE L*C*H* color space.

Chroma refers to the chromaticity coordinates which is the perpendicular distance from the lightness (Desalegn and Hailu 2020). Chroma values increased with an increment in the oyster mushroom percentages. Similar trends of hue angle and chroma values were observed in composited flour samples with an increment in levels of anchote flour.

The current research findings collaborates with that of Abou‐Zaid et al. (2012) in which increment in levels of the milled mushroom micelles to wheat flour led to a slight increment in L*, b*, chroma and hue angle values in the composited flours.

### 3.4 Dynamic Rheological Properties of the Prepared Thin Porridges

**FIGURE 2:**
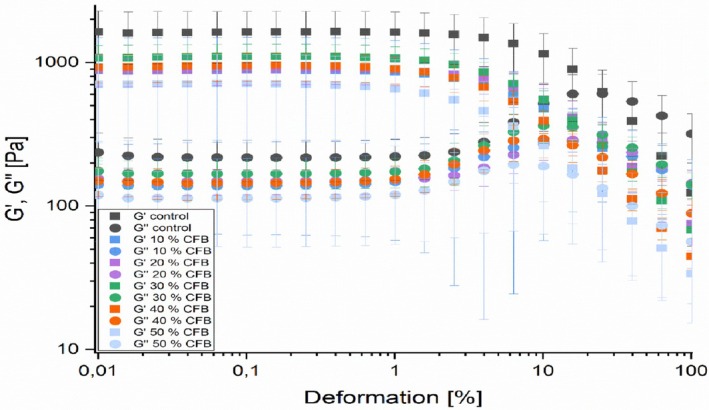
Loss and storage modulus (amplitude sweep) of sorghum‐pearl millet and oyster mushroom composite flours.

**FIGURE 3:**
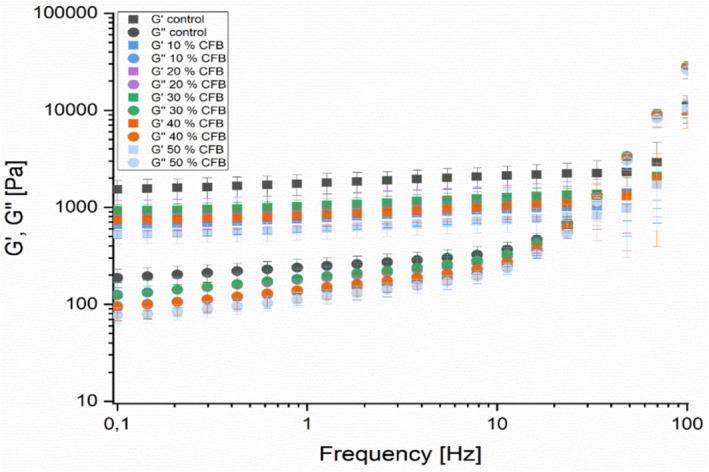
Loss and storage modulus (frequency sweep) of sorghum‐pearl millet and oyster mushroom composite flours.

The viscoelastic characteristics of the composite flour samples obtained by rheometer are presented in Figures 2 and 3.

Figure 2 shows the typical results of amplitude sweeps, on the *x*‐axis the amplitude strain (*γ*) is plotted, on the *y*‐axis both the loss and storage modulus are plotted with both on a logarithmic scale. It is noticeable that an increment in both curves from being linear before their decrease, an indication that an increase in energy proportion of the deformation (loss modulus *G*″) is being utilized to modify the structure before final breakdown happens. Increasing *G*′ values curves could be counter in maintaining the structure from increased energy of proportion of deformation. Additionally, in Figure 1 it can be noted that thin porridges from the composited flour samples had a gel like character *G*′ > *G*″ under the conditions of measurement meaning elastic behavior prevailed over the viscous behavior.

In the amplitude sweep graphs, the loss and storage curves showed constant higher plateau values if the strain amplitude remained below the limiting value; therefore, the limit of the linear viscoelastic region (LVE) limit range was exceeded at higher amplitudes. For all CFB in this study, LVE region can be defined from 0.01% to 1%. There seems to be no influence of substitution levels on the LVE region. After passing the γl (limiting value), sample structures had already been changed irreversibly or even destroyed. The amplitude sweeps storage and loss modulus graph values decreased with increase in oyster mushroom percentages, therefore both the *G*′ and *G*″ graphs values were high in control and low in composite flour with 50% oyster mushroom, although for all the composited flour samples *G*′ values were greater than those for *G*″, an indication that the composite flour samples elastic behavior (gel like character) prevailed over viscous behavior.

In Figure 3, the loss and storage modulus are plotted versus frequency (Hz). Elastic or transition to flow region is the first noticeable change, because when viewed from higher frequencies it is where elastic behavior dominates, *G*″ describing viscous behavior becomes significant (van de Ven 1985). The plateau or rubbery region which is a region at angular frequencies (0.1–10 1/s), elastic behavior dominated in this region over the viscous behavior *G*′ > *G*″ and hence values of loss modulus were lower when compared to storage modulus hence all the composited flour thin porridges had structure of gel at this region.

From plateau region the graph shifts to higher transition crossover or what is known as leathery region which appears between angular frequencies (10–100 1/s). Loss modulus value at this region rises faster than storage modulus due to high relaxation frequency and dissipation mechanisms (van de Ven 1985). At *G*′ = *G*″ which is known as crossover frequency, measures the longest time of relaxation. At higher angular frequencies than 100 1/s the glassy region appears where the *G*″ continues to predominate. Therefore, from the dynamic oscillatory measurements it can be concluded that at the LVE region both loss and storage modulus are independent of amplitude strain.

The oscillatory findings are in collaboration with Ahmed (2012), where lupine flour addition to dough caused an increase in the dough elasticity. Similarly, by increasing cassava proportion in composite flour samples from cereals resulting in stiff porridges showed a reduction in both loss and storage modulus, although the elasticity increased *G*′ > *G*″ (Bangu et al. 2000). The results of the rheological tests underline the findings of the pasting tests as the low viscosity levels maintained after cooking and no further swelling appears.

## 4 Conclusions

This research intended to investigate the color, pasting, functional properties of composite flour blends and rheological properties of the composited flour thin porridges from pearl millet‐sorghum flour fortified with oyster mushroom. It was stated that they contribute directly to the product quality of pearl millet‐sorghum and oyster mushroom composite flour products (thin porridge) not only regarding the cooking quality. This research has therefore elicited the information on the influence of these properties on the product. Therefore, for the functional properties results it can be concluded that, incorporation of oyster mushrooms led to a decrease in bulk density, increased swelling properties and both oil and water absorption hence inducing a positive impact on the thin porridges. The color change of the flours with increase in oyster mushroom levels (L*, b*, chroma, hue increased while a* decreased) could have effects on consumer acceptability of the porridges based on color preferences. The decrease in pasting parameters (PV, TV, BV, FV and SV) with increase in oyster mushroom level might influence the thin porridges viscosity, due to dilution of carbohydrate contents and small sugars and hence affect consumer perception on the products. In both the amplitude and the frequency sweeps *G*′ prevailed over the *G*″ and therefore suggesting that the thin porridges appeared more elastic than viscous (*G*′ > *G*″), also an increment in both the *G*′ and *G*″ values with increase in strain, although increasing oyster mushrooms amounts led to a decline in moduli values, suggesting that the behaviors could have also been a contributing factor to the decrease in their acceptability. Therefore, according to the present and prior results the target population of the flours should be the young children from six years to adolescents although, the evaluation of protein digestibility and the structural properties of the flours were not done thus this comes as the limitation of the study and therefore further research should be done in order to better understand the possible uses of the flours.

## Author Contributions

**Alice Ndunge Charles:** conceptualization (equal), data curation (equal), formal analysis (equal), methodology (equal), software (equal), visualization (equal), writing – original draft (equal). **Monica Mburu:** conceptualization (equal), funding acquisition (equal), project administration (equal), resources (equal), supervision (equal). **Daniel Njoroge:** investigation (equal), supervision (equal), validation (equal), writing – review and editing (equal). **Mario Jekle:** visualization (equal), writing – review and editing (equal). **Viktoria Zettel:** data curation (equal), formal analysis (equal), funding acquisition (equal), project administration (equal), resources (equal), supervision (equal), writing – review and editing (equal).

## Funding

This work was financially supported by Dedan Kimathi University of Technology Graduate Assistantship Program, National Research Fund Kenya (NRF‐K) under CHIAM project and German Federal Ministry of Food and Agriculture (BMEL) through office for Agriculture and Food (BLE) grant number 2821ERA19C. This project received funding from the European Union's Horizon 2020 Research and Innovation Program under grant agreement No 862555, the work has been performed within the ERA‐NET “FOSC”.

## Ethics Statement

The approval of the study was done by Dedan Kimathi University of Technology (DeKUT) school of graduate studies and research after ethical considerations (REF: DeKUT/SGSR/SF/B214) on 20th July, 2022.

## Conflicts of Interest

The authors declare no conflicts of interest.

## Data Availability

Data that support the findings of this study are openly available https://link.springer.com/article/10.1007/s44187‐024‐00219‐z.
